# Real‐world outcomes of anti‐EGFR therapy in advanced non–small cell lung cancer EGFR mutated in Peru

**DOI:** 10.1111/1759-7714.14714

**Published:** 2022-11-11

**Authors:** Marco Galvez‐Nino, Rossana Ruiz, Katia Roque, Ofelia Coanqui, Natalia Valdivieso, Mivael Olivera, Apar Kishor Ganti, Luis Mas

**Affiliations:** ^1^ Department of Medical Oncology Instituto Nacional de Enfermedades Neoplásicas Lima Peru; ^2^ Escuela Profesional de Medicina Humana Universidad Privada San Juan Bautista Lima Peru; ^3^ Universidad Científica del Sur Lima Peru; ^4^ VA Nebraska Western Iowa Health Care System, University of Nebraska Medical Center Omaha Nebraska USA; ^5^ Universidad Peruana Cayetano Heredia Lima Peru

**Keywords:** EGFR, Latin American, lung cancer, real‐world, survival

## Abstract

**Background:**

Despite the advances in the management of advanced non–small cell lung cancer (NSCLC), the access to genetic profiling and target therapies remains a challenge in Latin America, even in countries with a higher rate of targetable mutations. The aim of this study is to evaluate the clinical outcomes of anti‐epidermal growth factor receptor (EGFR) tyrosine kinase inhibitors (TKI) treatment in a Peruvian real‐world setting.

**Methods:**

This is a retrospective study of recurrent or advanced NSCLC EGFR mutated patients diagnosed and treated with anti‐EGFR TKI at Instituto Nacional de Enfermedades Neoplásicas (INEN) between January 1, 2015 to December 31, 2020. The outcomes were objective response rate (ORR), progression free survival (PFS), and overall survival (OS).

**Results:**

We identify 613 stage IV or recurrent NSCLC patients who were tested for EGFR mutations and found a pathogenic mutation in 39.5% of patients. Only 51.2% of them received anti‐EGFR TKI as institutional treatment. ORR was 58%, after median follow‐up of 32 months, the estimated median PFS was 13.9 months (11.1–16.7 months), and the estimated median OS was 21.7 months (18.5–24.9 months). No differences were found in PFS according to line of treatment or brain metastases at diagnosis (*p* = 0.46 and *p* = 0.07, respectively), respect to OS there were no differences line of treatment (*p* = 0.12), significant difference were found in presence of brain metastases (*p* = 0.006).

**Conclusion:**

Our study demonstrates that erlotinib for advanced NSCLC harboring EGFR‐activating mutations is effective even in patients usually excluded from clinical trial, like those previously exposed to one or more lines of chemotherapy or with brain metastases.

## Graphical abstract

After molecular study of 613 patients with stage IV or recurrent non–small cell lung cancer, epidermal growth factor receptor (EGFR) mutation was detected in 39.5% of patients, however, only 51.2% received anti‐EGFR tyrosine kinase inhibitors as institutional treatment and 72 (0.6%) of them as first line. Despite the lack of access to target therapy and the late onset of treatment, erlotinib showed effectiveness even in patients previously exposed to one or more lines of chemotherapy or with brain metastases.

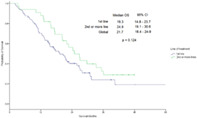

## INTRODUCTION

Genetic profiling and targeted therapy have revolutionized the management of patients with advanced non–small‐cell lung cancer (NSCLC), generating a significant survival improvement in these patients compared to what was achieved with traditional cytotoxic therapy.^1^, ^2^, ^3^ Despite advances in the management of this disease, it continues to be the leading cause of cancer‐related death worldwide, especially in countries with limited access to health services, such as Latin American countries.^4^, ^5^ In Peru, lung cancer is the second cause of cancer deaths with survival rates lower to 10% at 5 years.^6^

In this context, one of the most frequent actionable mutations is related to the (epidermal growth factor receptor) EGFR gene; this mutation represents 10% to 15% of cases in Caucasian patients and even more than 50% in some reports of East Asian patients.^7^, ^8^, ^9^ However, multiple reports indicate that in Peru, this mutation occurs in between 32% and 39% of patients with advanced NSCLC, a higher frequency compared to other countries in the region.^10^, ^11^, ^12^

Several clinical trials have shown that EGFR tyrosine kinase inhibitors (TKI) confer a higher clinical benefit compared to chemotherapy,^13^, ^14^, ^15^, ^16^ positioning itself as the recommended standard of care worldwide. Despite this, the access to innovative treatment and molecular tests required for its use remains a limitation in many Latin American countries.^5^

The Instituto Nacional de Enfermedades Neoplásicas (INEN) is the main referral public institution for cancer treatment in Peru and its services are covered mainly by the national subsidized insurance regimen. In this setting, it is the only public center that performs molecular detection of EGFR mutations and one of the first to have access to targeted therapy for this disease.^17^ Here, we describe the real‐world outcomes of the use of erlotinib in NSCLC EGFR mutated patients.

## MATERIALS AND METHODS

### Study design and patients

This is a retrospective single‐institution study that included recurrent or advanced NSCLC EGFR mutated patients diagnosed at INEN between January 1, 2015 to December 31, 2020. Inclusion criteria were: (1) histological confirmed diagnosis of NSCLC, (2) available result of EGFR test performed in stage IV or recurrent disease setting, and (3) record of erlotinib use as institutional treatment for NSCLC EGFR mutated patients.

### Study procedures and outcomes

We collected demographic and clinical features, including age, sex, smoking status, exposure to wood smoke, Eastern Cooperative Oncology Group (ECOG) performance status, presence of brain metastases at diagnosis, and EGFR mutation information of patients who received erlotinib as institutional treatment. For the analysis of effectiveness, we stratified patients by presence or absence of brain metastases at diagnosis and line of treatment, classified as (1) first line of treatment defined as patients without exposure of any chemotherapy treatment or up to three previous cycles of chemotherapy without disease progression and (2) second or more lines of treatment. Progression free survival (PFS) was defined as the time from the beginning erlotinib use until progression, death, or lost to follow‐up. Overall survival (OS) was defined as the time from starting erlotinib to death. If date of death was not registered, vital status was obtained from the National Registry of Identification and Civil Status. Survival curves were constructed with the Kaplan–Meier method. The statistical package used for data analysis was SPSS 19.0 (IBM). Information was extracted of medical records after the approval by the Institutional Review Board from INEN.

## RESULTS

### Patients and EGFR status

During the study period, we identified 613 stage IV or recurrent NSCLC patients who were tested for EGFR mutations. Of these patients, 124 received erlotinib as institutional treatment and were included in the main analysis. The clinical characteristics of them are shown in Table 1. The median age was 60 years (range, 25–81 years), 66.1% were women, 18.5% were smokers, 23.4% had exposition to wood smoke, 71% were ECOG 1, and 28.2% had brain metastases at diagnosis.

**TABLE 1:** Clinical characteristic of patients treated with erlotinib

	*n* = 124, (%)
Median age (year, range)	60 (25–81)
Sex	
Male	42 (33.9)
Female	82 (66.1)
Smoker	
Yes	23 (18.5)
No	96 (77.4)
Unknown	5 (4.0)
Wood smoke	
Yes	29 (23.4)
No	69 (55.6)
Unknown	26 (21.0)
Performance status (ECOG)	
1	88 (71.0)
2	30 (24.2)
3	6 (4.8)
Brain metastases	
Yes	35 (28.2)
No	89 (71.8)
Erlotinib use	
1st line	90 (72.6)
2nd or more line	34 (27.4)

Abbreviations: ECOG, Eastern Cooperative Oncology Group.

**FIGURE 1:**
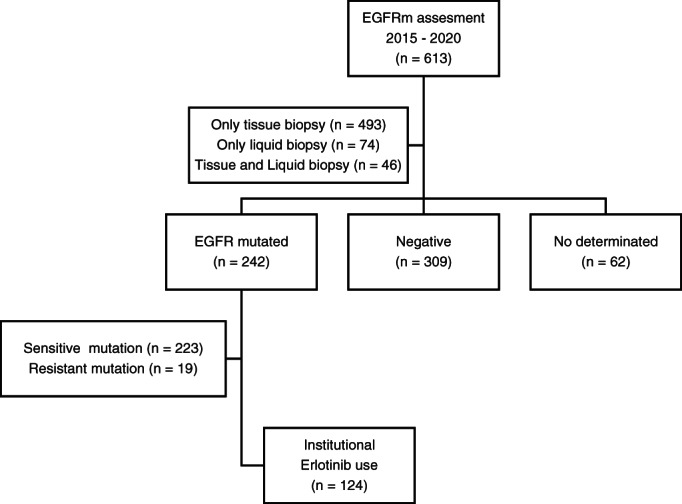
Patient selection flow chart.

**FIGURE 2:**
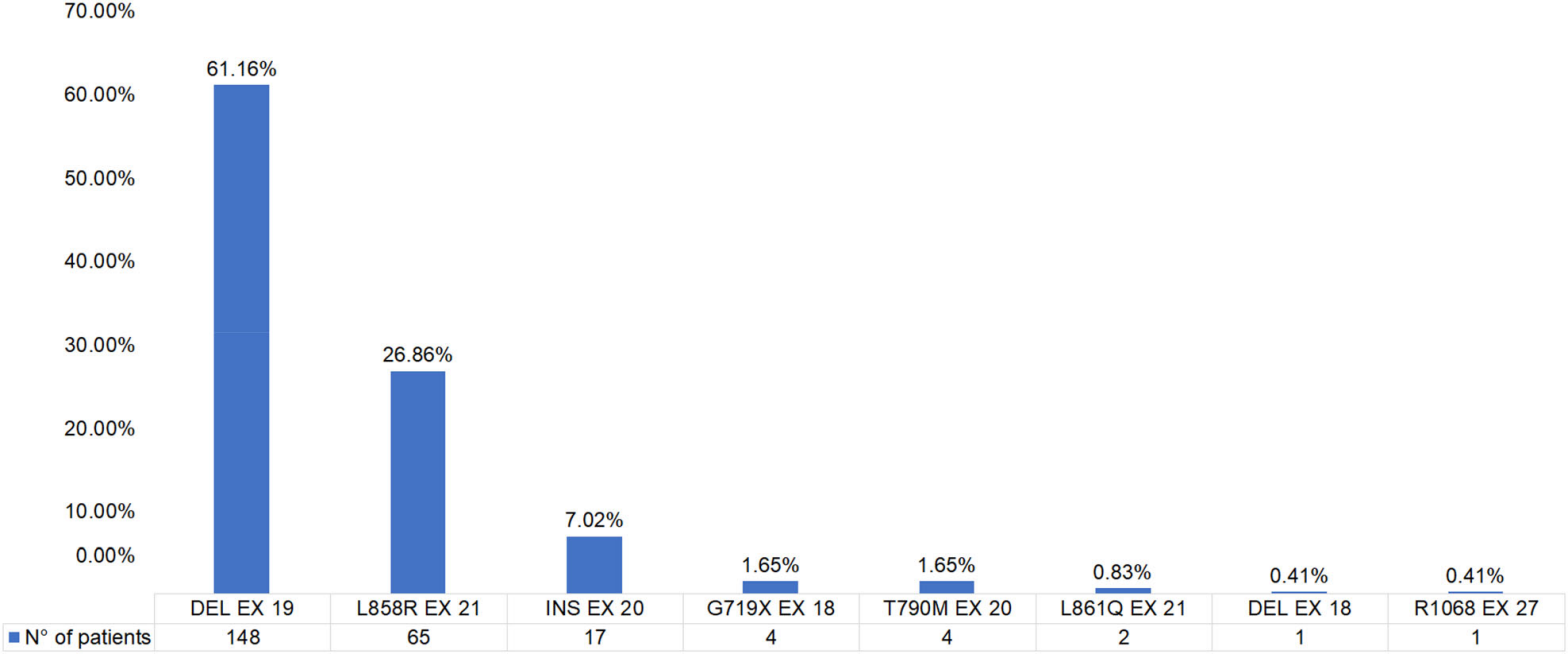
Frequency of epidermal growth factor receptor mutations.

The presence of EGFR mutation was performed by institutional polymerase chain reaction (PCR) in tissue biopsy (*n* = 539) and by non‐institutional next‐generation sequencing (NGS) liquid biopsy (*n* = 121). Pathological EGFR mutations was found in 39.5% of patients (*n* = 242) and of them 213 were common mutations (Figure 1). The most frequent of mutations were deletions in Exon 19 (61.16%), followed by the L858R mutation (26.86%), and insertions in Exon 20 (7.02%) (Figure 2). In the 61 patients whose EGFR mutation was detected by NGS, the most frequent concurrent genetic alterations were TP53 (70.5%), CTNNB1 (6.6%), and NF1 (6.6%).

### Outcomes

**FIGURE 3:**
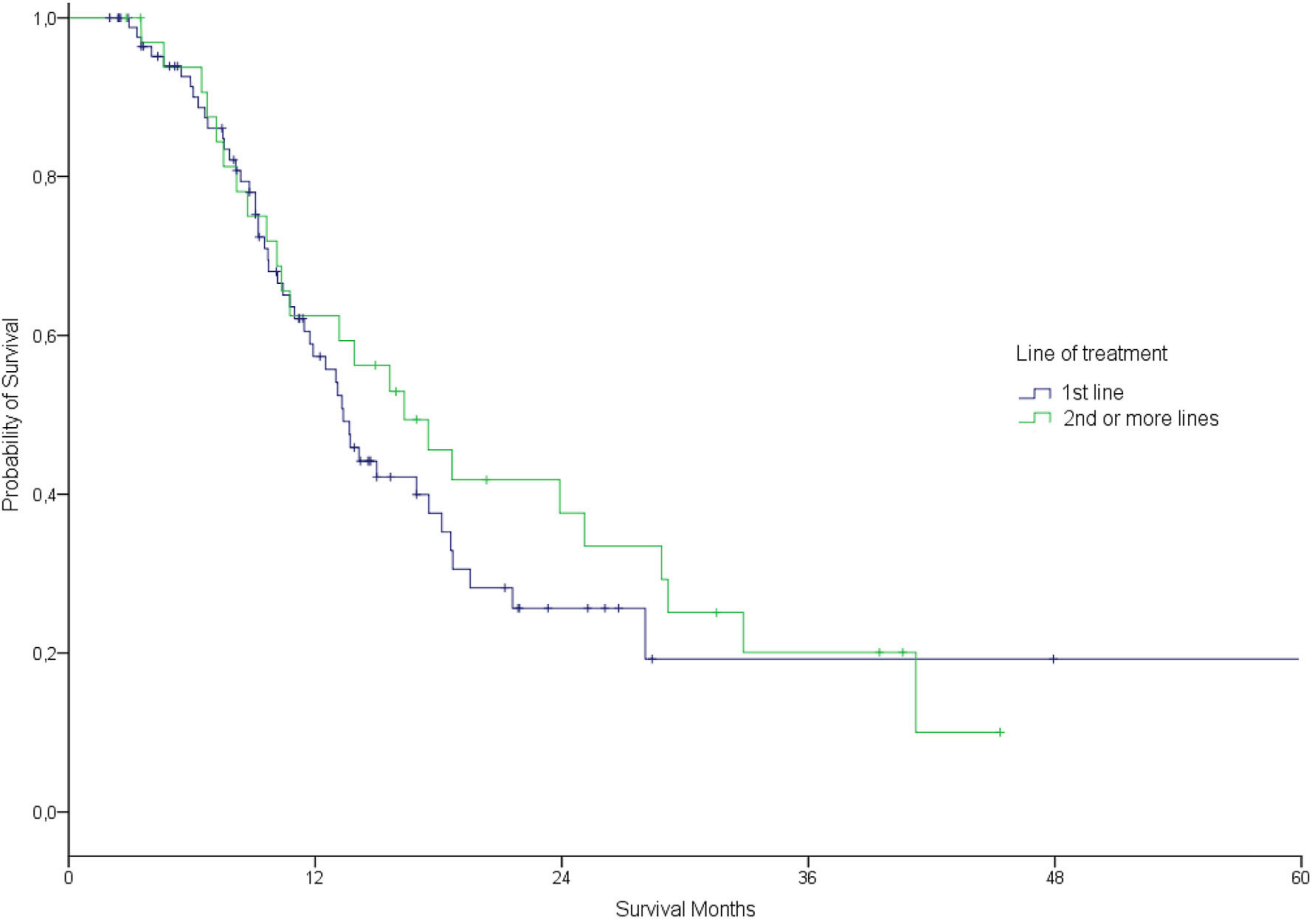
Estimated progression free survival by line of tyrosine kinase inhibitors treatment 1st versus 2nd or more (long‐rank *p* = 0.46).

**FIGURE 4:**
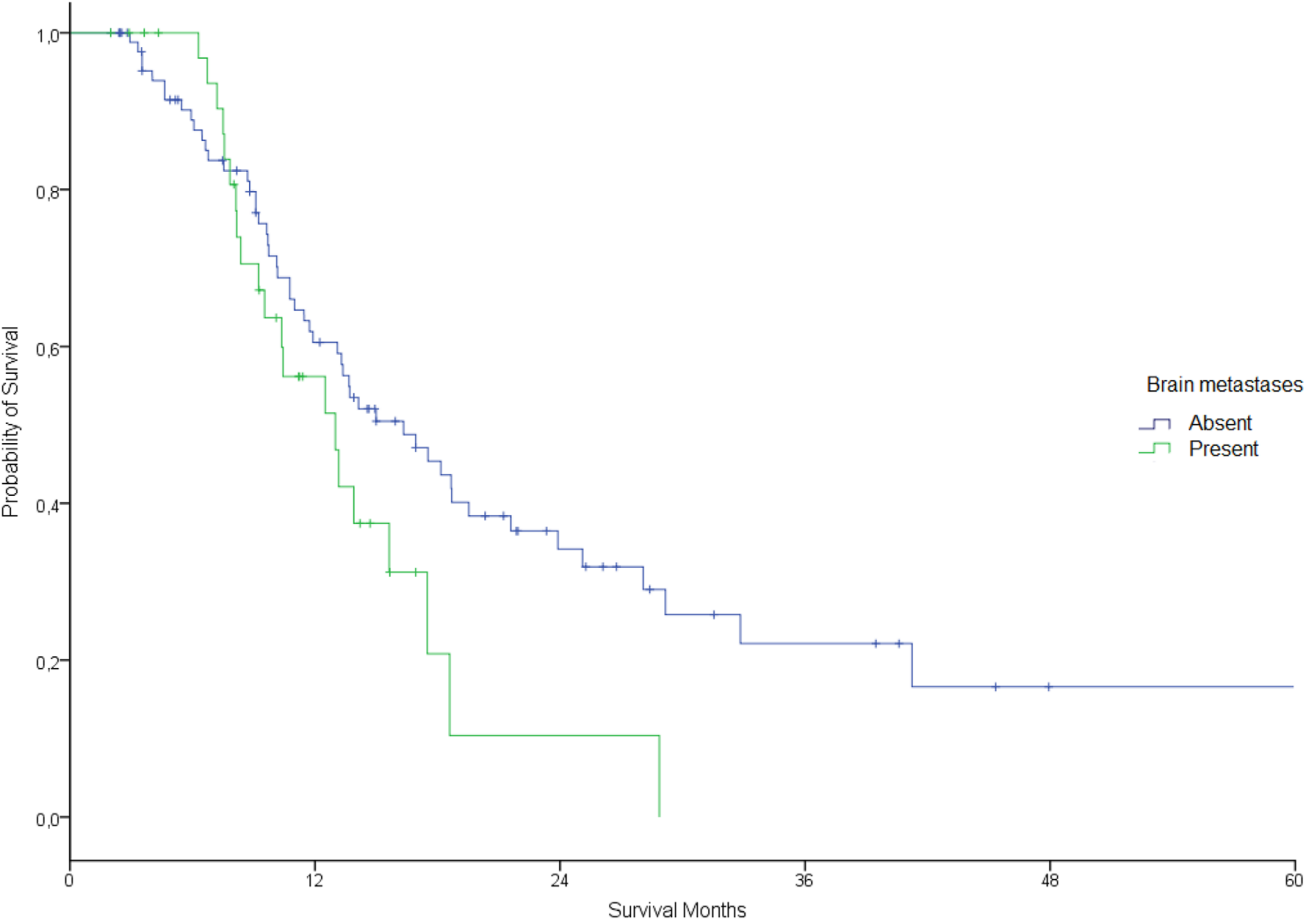
Estimated progression free survival by presence versus absence of brain metastases at diagnosis (long‐rank *p* = 0.07).

**FIGURE 5:**
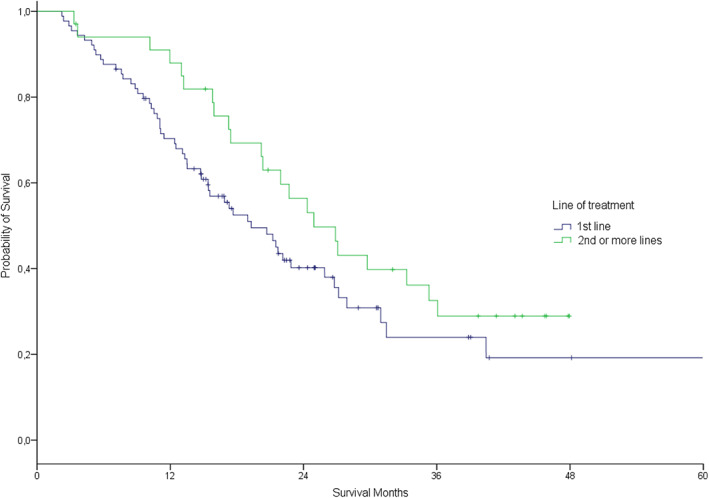
Estimated overall survival by line of tyrosine kinase inhibitors treatment 1st versus 2nd or more (long‐rank *p* = 0.12).

**FIGURE 6:**
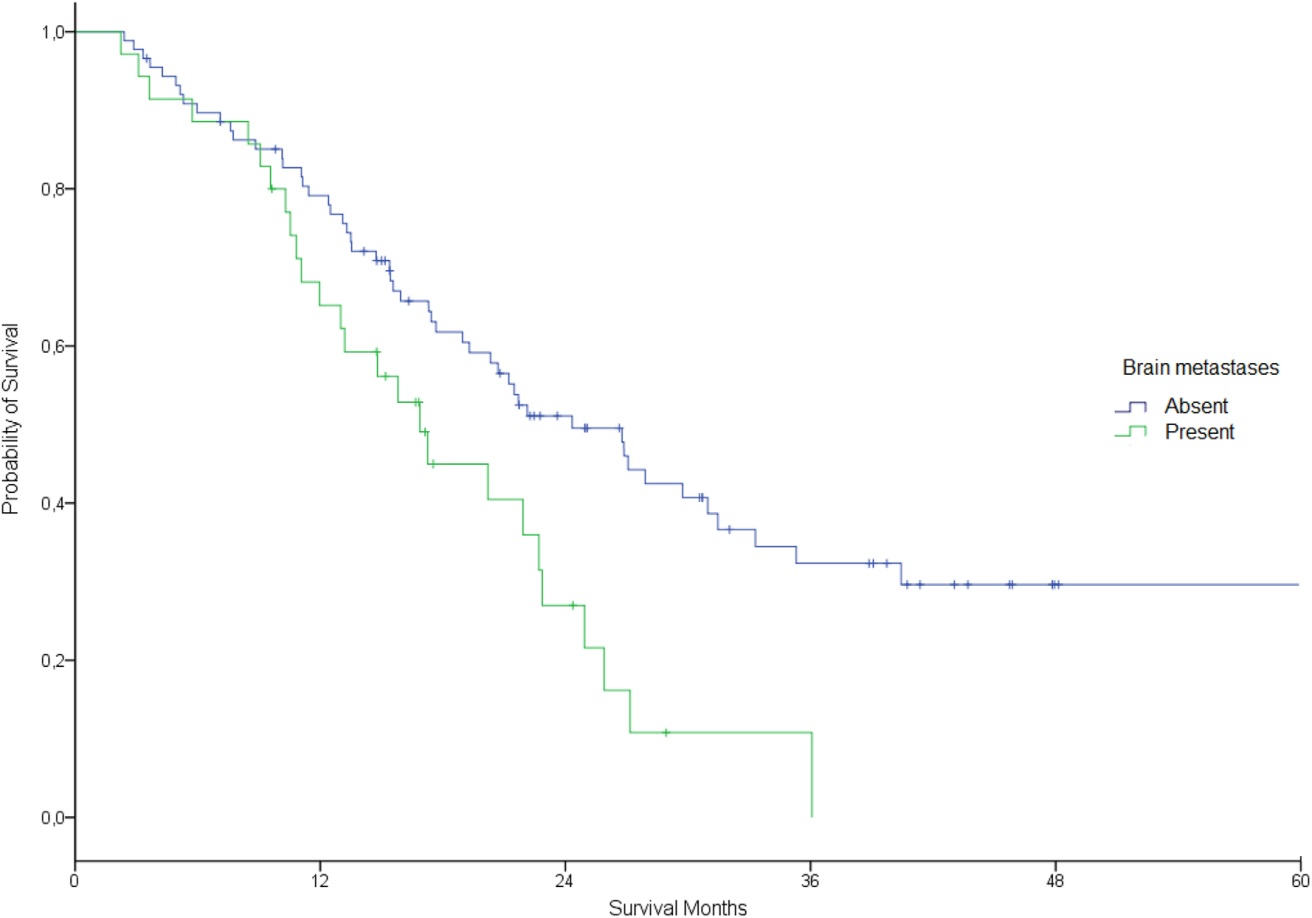
Estimated overall survival by presence versus absence of brain metastases at diagnosis (long‐rank *p* = 0.006).

The objective response rate was 58%, with six (4.8%) patients achieving complete response and 66 (53.2%) partial response (Table 2). After median patient follow‐up of 32 months (95% CI, 23.7–40.2), 76 patients had progressed. Of these, 40 (52.8%) were tested for T790M resistance mutation and 21 (55.3%) were positive. The estimated median PFS was 13.9 months (11.1–16.7 months) for all patients. There was no difference between first line and second or more line of treatment group (13.4 vs. 16.3 months, *p* = 0.46) (Figure 3), and neither between presence or absence of brain metastases (13.0 vs. 16.3 months, *p* = 0.07) (Figure 4). Estimated median OS from the beginning of TKI treatment was 21.7 months (18.5–24.9 months) for all patients, without any difference between first line and second or more line of treatment group (19.3 vs. 24.9 moths, *p* = 0.12) (Figure 5), but finding differences according to the presence or absence of brain metastases (16.8 vs. 24.3 months, *p* = 0.006) (Figure 6).

**TABLE 2:** Response rate according line of treatment

	Total patients (*n* = 124), No. (%)	1st line	2nd or more
Complete response	6 (4.8)	5 (5.6)	1 (2.9)
Partial response	66 (53.2)	49 (54.4)	17 (50.0)
Stable disease	30 (24.2)	17 (18.9)	13 (38.2)
Progressive disease	3 (2.4)	3 (3.3)	–
Unknown	19 (15.3)	16 (17.8)	3 (8.8)
Overall response rate	72 (58.0)	54 (60.0)	18 (52.9)

## DISCUSSION

EGFR mutations represent one of the most common pathogenic genomic alteration in NSCLC patients, however, in the Latin American setting it has a heterogeneous frequency, for example, countries such Peru, Mexico, and Costa Rica have a high frequency of EGFR, similar to Asian populations, and countries such Argentina, Chile, and Uruguay have a frequency similar to North American and European population.^10^, ^18^ In this study, as in previous reports,^10^ the rate of EGFR mutated patients was high (39.5%), this finding could be associated with the low rate of smokers^19^ and is possibly related with the genomic ancestry of Peruvians that is close to the Asian population.^11^, ^12^

Despite the higher frequency of EGFR mutations and other pathogenic and targetable mutations in Latin American NSCLC patients, the access to molecular assays and target therapies remain a limitation,^20^ especially in countries like Peru where there is a great fragmentation of its health system.^17^ The molecular profiling based on tissue samples is available in our institution since 2015; however, it is the only center in the network of institutions of the Peruvian Ministry of Health that has this technology. The determination rate of EGFR mutations status in this study was near to 90%, but 19.5% of patients required a liquid biopsy because of lack of either tissue sample or enough DNA. However, assays like this are not available in public institutions and were performed through industry testing support programs or clinical trials.

Although it is well known that the use of target therapy in patients who carry a targetable mutation significantly increases the survival compared to the use of chemotherapy, in our study only 55.6% of patients with a sensitive EGFR mutation received an institutional target treatment due mainly by the lack of access to TKI, the poor status performance at diagnosis, and the loss of follow‐up especially observed before 2017. It is well known that receiving this type of treatment according to guidelines remains a challenge even in developed countries because of different reasons,^21^, ^22^ however, the regulatory approval process, the cost of treatments, and the process of diagnosis are the main challenges in Latin America.^5^, ^23^ In Peru, targeted therapy against EGFR sensitive mutations was available in the public health system since 2017, which represents a delay in public access of 9 years since National Regulatory Approval and 13 years since Food and Drug Administration approval.^17^

Despite late onset of targeted therapy in a substantial proportion of patients observed in this study (27% of patients started TKI in second line or more), the survival achieved were comparable to those reported in clinical trials and other real‐world reports,^14^, ^24^, ^25^, ^26^ and we did not find any difference between survival from baseline of the TKI, regardless of line of TKI treatment (first vs. second or more). Of the group of patients who progressed, T790M mutation could be evaluated in only 52% of patients and was detected in 55% of cases; despite the percentage of patients evaluated, the detection rate was similiar to that previous reported.^27^ However, most of them could not receive osimertinib as a subsequent line of treatment because of limited availability in Peruvian public health system.^17^

The benefit of target therapy was also evidenced in prolonged PFS regardless the presence of brain metastases; however this benefit was not reflected in OS in this poor prognostic setting.

## CONCLUSIONS

The results of our study reinforce the evidence that targeted therapies for the treatment of diseases with targetable mutations provide a relevant benefit even if it is used beyond the first line of treatment or in presence of brain metastases. Therefore, efforts must be continue made to improve the access to molecular profiling and target therapies.

## CONFLICT OF INTEREST

This research did not receive any specific grant from funding agencies in the public, commercial, or not‐for‐profit sectors.
